# Laminar Organization of the Entorhinal Cortex in Macaque Monkeys Based on Cell-Type-Specific Markers and Connectivity

**DOI:** 10.3389/fncir.2021.790116

**Published:** 2021-12-07

**Authors:** Shinya Ohara, Rintaro Yoshino, Kei Kimura, Taichi Kawamura, Soshi Tanabe, Andi Zheng, Shinya Nakamura, Ken-ichi Inoue, Masahiko Takada, Ken-Ichiro Tsutsui, Menno P. Witter

**Affiliations:** ^1^Laboratory of Systems Neuroscience, Graduate School of Life Sciences, Tohoku University, Sendai, Japan; ^2^PRESTO, Japan Science and Technology Agency (JST), Tokyo, Japan; ^3^Systems Neuroscience Section, Department of Neuroscience, Primate Research Institute, Kyoto University, Inuyama, Japan; ^4^Laboratory of Systems Neuroscience, Graduate School of Medicine, Tohoku University, Sendai, Japan; ^5^Department of Developmental Neuroscience, Graduate School of Medicine, Tohoku University, Sendai, Japan

**Keywords:** medial entorhinal cortex, lateral entorhinal cortex, primates, calbindin, reelin, Purkinje cell protein 4, telencephalic projections

## Abstract

The entorhinal cortex (EC) is a major gateway between the hippocampus and telencephalic structures, and plays a critical role in memory and navigation. Through the use of various molecular markers and genetic tools, neuron types constituting EC are well studied in rodents, and their layer-dependent distributions, connections, and functions have also been characterized. In primates, however, such cell-type-specific understandings are lagging. To bridge the gap between rodents and primates, here we provide the first cell-type-based global map of EC in macaque monkeys. The laminar organization of the monkey EC was systematically examined and compared with that of the rodent EC by using immunohistochemistry for molecular markers which have been well characterized in the rodent EC: reelin, calbindin, and Purkinje cell protein 4 (PCP4). We further employed retrograde neuron labeling from the nucleus accumbens and amygdala to identify the EC output layer. This cell-type-based approach enabled us to apply the latest laminar definition of rodent EC to monkeys. Based on the similarity of the laminar organization, the monkey EC can be divided into two subdivisions: rostral and caudal EC. These subdivisions likely correspond to the lateral and medial EC in rodents, respectively. In addition, we found an overall absence of a clear laminar arrangement of layer V neurons in the rostral EC, unlike rodents. The cell-type-based architectural map provided in this study will accelerate the application of genetic tools in monkeys for better understanding of the role of EC in memory and navigation.

## Introduction

The entorhinal cortex (EC), a major gateway between the hippocampus and telencephalic structures, plays a critical role in memory and navigation. Previous anatomical studies have shown that the connectivity patterns of EC are strikingly different across the layers: superficial layers (layers II and III) provide the main inputs to the hippocampus, whereas deeper layers (layers V and VI) receive outputs from the hippocampus. These patterns are consistent in several species, including rodents and monkeys (Witter et al., 2017). In rodents, through the use of various molecular markers and genetic tools, it has further been established that entorhinal neurons constituting each layer exhibit distinct molecular phenotypes and hodological features (Witter et al., 2017; Kobro-Flatmoen and Witter, 2019). Principal neurons in EC layer II are of two types, stellate-like neurons and pyramidal neurons, the former of which express reelin, whereas the latter include a large population of calbindin-expressing neurons (RE+ and CB+, respectively). The RE+ neurons possess the typical projection pattern of EC layer II neurons, innervating the dentate gyrus and the CA3/CA2 regions of the hippocampus (Varga et al., 2010). In contrast, the CB+ excitatory neuron population comprises neurons with diverse projections (Ohara et al., 2019), targeting the stratum lacunosum of CA1 (Kitamura et al., 2014), ipsi- and contralateral EC (Varga et al., 2010; Zutshi et al., 2018), medial septum (Fuchs et al., 2016), and olfactory structures (Leitner et al., 2016). Principal neurons in EC layer III express Purkinje cell protein 4 (PCP4) and project to CA1 and the subiculum (Tang et al., 2015). In addition to these superficial layers, recent studies have demonstrated the organization of layer V (Sürmeli et al., 2015; Ohara et al., 2018, 2021; Rozov et al., 2020). In EC layer V, PCP4 is also a useful marker for identifying a subpopulation of excitatory neurons. Neurons in the deep-sublayer Vb contain PCP4 and chicken ovalbumin upstream promoter transcription factor (COUP-TF) interacting protein 2 (Ctip2), and are the main recipient of hippocampal inputs. In contrast, neurons in the superficial-sublayer Va express E twenty-six variant1 (Etv1), and are the major origin of EC outputs. Although EC layer VI neurons are still understudied, it is evident that cell-type-specific approaches with genetic tools have immensely advanced our understandings of the organization of complicated entorhinal circuits and their functions (Kitamura et al., 2014, 2015, 2017; Kobro-Flatmoen et al., 2016; Leitner et al., 2016; Kanter et al., 2017; Rowland et al., 2018; Zutshi et al., 2018; Vandrey et al., 2020; Lee et al., 2021).

The EC in various species has been divided into cytoarchitectonically identified subregions of which number has been variable, though often include two main anatomically and functionally defined subdivisions, the lateral and the medial entorhinal cortex (LEC and MEC, respectively; Stephan, 1975; Witter et al., 1989, 2017). The functional differences of these two entorhinal subdivision have been examined mainly in rodents. Activity of neurons in MEC is spatially modulated, reflecting self-location relative to the geometry of the environment. By contrast, such spatial modulation is essentially absent from LEC neurons, activity of which is associated with odors or objects in context (Fyhn et al., 2004; Deshmukh and Knierim, 2011; Neunuebel et al., 2013; Tsao et al., 2013; Moser et al., 2014; Gofman et al., 2019). Interestingly, the distribution of the molecularly defined neuron types described above differs in these two subdivisions. A prominent difference between MEC and LEC can be observed in EC layer II of rodents (both rats and mice). In MEC, RE+ and CB+ neurons are intermingled in layer II, and CB+ neurons appear to be grouped in patches (Varga et al., 2010; Kitamura et al., 2014; Ray et al., 2014). In LEC, on the other hand, the two types of neurons form separate sublayers, a superficial-sublayer enriched with RE+ cells and a deeper-sublayer enriched with CB+ cells (Leitner et al., 2016; Ohara et al., 2019). Whether to call this CB+ sublayer in LEC “superficial layer III” or “layer IIb” differs depending on the schemes for delineating layer II/III in rodent LEC (see Kobro-Flatmoen and Witter, 2019 for a more elaborate description). In the original definition by Cajal and Lorente de Nó, the stellate-like neurons were included in layer II, whereas the pyramidal neurons immediately deep to the stellate cells were considered to make up the superficial part of layer III. This “traditional” laminar differentiation thus leads to a discrepancy, in that CB+ cells constitute part of layer II in MEC and part of layer III in LEC. In contrast, the other scheme in this study, we opt to follow the more recent description that LEC layer II includes the CB+ pyramidal neurons as in the case for MEC, by defining RE+ sublayer as layer IIa and CB+ sublayer as layer IIb.

The two EC subdivisions further differ slightly in layer III, since PCP4 labeling is more prominent in MEC than in LEC (Ohara et al., 2018). The organization of layer V, however, is rather similar in MEC and LEC of rodents, given that this layer is composed of a PCP4/Ctip2-positive deep sublayer and a superficial sublayer where these two markers are virtually absent (Ohara et al., 2018, 2021). Although the molecular markers for layer V have only been identified recently (Sürmeli et al., 2015; Kitamura et al., 2017; Ohara et al., 2018), the laminar definition of layer V itself is in line with previous studies based on cytoarchitectonic features (Insausti et al., 1997; Hamam et al., 2000, 2002; Canto and Witter, 2012a,b).

In monkeys, EC has been organized into the varying number of cytoarchitectonically defined subdivisions, and a parcellation is now widely used to divide EC into seven parts: the olfactory field (Eo), rostral division (Er), lateral rostral division (Elr), lateral caudal division (Elc), intermediate division (Ei), caudal division (Ec), and caudal-limiting division (Ecl) (Amaral et al., 1987; Piguet et al., 2018). Alternatively, EC has also been subdivided into two subregions, which correspond to the rodent LEC and MEC, based on both the architectonic features (for review see Amaral et al., 1987) and connectivity with the hippocampus and parahippocampal regions (Witter and Amaral, 1991; Suzuki and Amaral, 1994). According to these connectivity studies, it is surmised that monkey LEC comprises Eo, Er, Elr, and rostral Ei, whereas monkey MEC comprises caudal Ei, Elc, Ec, and Ecl. However, the precise border between the two rostrocaudally separate portions within Ei has not yet been clearly identified (Witter and Amaral, 2020). In addition to these anatomical studies, recent human studies using functional connectivity MRI have further suggested that the rostrolateral and caudomedial EC might represent areas similar to rodent LEC and MEC, respectively (Maass et al., 2015; Schröder et al., 2015). Although functional connectivity is a powerful approach to identify functional analogy, it must be noted that anatomical approaches, eventually combined with developmental studies, are needed to decipher the homology of EC across species (Witter et al., 2017).

One potential index to clarify the homologous brain regions of LEC and MEC in monkey EC is cell-type-specific molecular markers. In monkeys, the immunoreactivity for RE in layer II has been reported (Martínez-Cerdeño et al., 2002), and that for CB has been examined in detail across all EC subdivisions (Suzuki and Porteros, 2002). Data on the distribution of PCP4+ neurons, on the other hand, have not so far been available in monkeys. The first aim of this study is to systematically investigate the distribution patterns of the molecular markers for excitatory EC neurons (i.e., RE, CB, and PCP4) in macaque monkeys and to determine whether the two EC subregions, homologous to rodent LEC and MEC, can be characterized by these molecular markers. Our second aim is to explore the organization of layer V in monkey EC. As described above, recent studies in rodents have shown that layer V is composed of layers Va and Vb, which exhibit distinct patterns of neuronal connectivity and gene expression (Sürmeli et al., 2015; Ohara et al., 2018, 2021). Whether monkey EC shares a similar organization of layer V to rodent EC remains unknown. To confirm the organization of monkey layer V, the distribution of neurons projecting to subcortical structures was examined by retrograde labeling technique. Although this is a semi-quantitative case study which is limited to investigating specific patterns in two monkeys, to the best of our knowledge, the present work provides the first cell-type-based global map of EC in macaque monkeys. This will accelerate the application of genetic tools in monkeys, and will bridge the gap in cell-type-specific understanding of EC organization between rodents and primates.

## Materials and Methods

### Experimental Animals

#### Monkeys

Two male macaque monkeys (Macaca mulatta) of 6 years old weighing 9.8 kg and 5 years old weighing 6.7 kg were used in this study (subjects Monkey-1 and Monkey-2, respectively). The experimental procedure for monkeys were in accordance with protocols approved by the Animal Welfare and Animal Care Committee of the Primate Research Institute, Kyoto University, and were conducted in line with the Guideline for Care and Use of Non-human Primates established by the Primate Research Institute, Kyoto University.

#### Rodents

Male adult Long Evans rats of 45–65 weeks weighing 320–390 g and male adult C57BL/6NCrSlc mice of 12–28 weeks weighing 18–33 g were used in this study. The experiments were approved by the Center for Laboratory Animal Research, Tohoku University, and were conducted according to the Guidelines of the National Institutes of Health and the Tohoku University Guidelines for Animal Care and use.

Throughout the experiments, the animals were maintained in a cage that was placed inside a safety cabinet. The room temperature (23–26°C) and the light condition (12 h on/off cycle) were controlled. All efforts were made to avoid the animals from suffering.

### Surgical Procedures

#### Monkeys

Monkeys were deeply anesthetized with ketamine (5.0 mg/kg, i.m.) and xylazine (0.5 mg/kg, i.m.). During the surgery, the monkey was kept hydrated with a located Ringer’s solution (i.v.). An antibiotic (Ceftazidime; 25 mg/kg, i.v.) and an analgesic (Meloxicam; 0.2 mg/kg, s.c.) were applied at the initial anesthesia. After removal of a portion of the skull, multiple injections of retrograde adeno-associated viral vector (AAV2Retro) (Tervo et al., 2016) were made unilaterally into the nucleus accumbens (NAc) and the amygdala (AMG) by the aid of a magnetic resonance imaging (MRI)-guided navigation system (Brainsight Primate, Rough Research, Canada). In total, 4.5 μl of the AAV2Retro-hSynI-AcGFP was injected into the NAc at two rostrocaudally different levels (2.0 × 10^13^ gc/ml, 1.5 μl × 3 site/2 track), and 4.8 μl of the AAV2Retro-hSynI-mKO2 was injected into the accessory-basal nucleus of AMG at two rostrocaudally different levels (2.0 × 10^13^ gc/ml, 1.2 μl × 4 site/2 track) through a 10 μl Hamilton microsyringe. After the injections, the scalp incision was sutured. The monkey was monitored until the full recovery from the anesthesia.

#### Rodents

Rats/mice were deeply anesthetized with ketamine (60 mg/kg, i.p.) and xylazine (4.8 mg/kg, i.p.) and were mounted in a stereotaxic flame. The skull was exposed, and a small burr hole was drilled above the injection site. The injection was made by means of a glass micropipette (tip diameter = 20–40 μm) connected to 1 μl Hamilton microsyringe. Coordinates of following injection sites were based on the rat or mouse brain atlas (Franklin and Paxinos, 2007; Paxinos and Watson, 2007) and calculated from bregma. For retrograde tracing experiments, rats/mice received an injection of 100–150 nl of 2.5% Fluoro-Gold (Fluorochrome) into the NAc at the rate of 25 nl per minute. The micropipette was left in place for an additional 15 min after the injection, before it was slowly withdrawn from the brain. When all injections were completed, the wound was sutured and the animal was monitored for recovery from anesthesia and returned to its home cage.

### Immunohistochemistry and Analysis

#### Monkeys

Three weeks after AAV injection, the monkeys were deeply anesthetized with an overdose of sodium pentobarbital (50–100 mg/kg, i.v.) and perfused transcardially with 0.1M phosphate buffer saline (PBS), followed by 10% formalin dissolved in 0.1M phosphate buffer (PB). The brain was removed from the skull, postfixed in the same fresh fixative overnight at 4°C, and saturated with 30% sucrose in 0.1 M PBS at 4°C. Monkey brains were sectioned in 50 μm coronal planes on a freezing microtome and 10 equally spaced series were collected. Sections were split in the mid-sagittal plane and processed as described below.

To visualize the PCP4+ neurons, sections ipsilateral to the injection site were stained with primary antibody, rabbit anti-PCP4 (1:250, Merck Millipore #HPA005792) followed by secondary antibodies (1:300, Alexa Fluor 647 goat anti-rabbit IgG, Jackson ImmunoResearch #111-605-144; 1:400, Alexa Fluor 488 donkey anti-rabbit IgG, Jackson ImmunoResearch, #711-545-152). To visualize RE+ and CB+ neurons, contralateral sections were stained with primary (1:250, mouse anti-Reelin (CR-50), MBL #D233-3; 1:1,000, rabbit anti-Calbindin, Abcam #ab11426) and secondary antibodies (1:400, Alexa Fluor 555 donkey anti-mouse IgG, Thermo Fisher scientific #A-315070; 1:300, Alexa Fluor 647 goat anti-rabbit IgG, Jackson ImmunoResearch #111-605-144; 1:400, Alexa Fluor 647 goat anti-mouse IgG, Jackson ImmunoResearch #115-605-003; 1:400, Alexa Fluor 555 goat anti-rabbit IgG, Jackson ImmunoResearch #111-165-144). For delineation purposes, sections were stained for NeuN with primary (1:1,000, mouse anti-NeuN, Merck Millipore #MAB377; 1:1,000, guineapig anti-NeuN, Merck Millipore #ABN90P) and secondary antibodies (1:400, Dylight 405 donkey anti-guineapig, Jackson ImmunoResearch #706-475-148; 1:400, Alexa Fluor 647 goat anti-mouse IgG, Jackson ImmunoResearch #115-605-003).

For all immunohistochemical procedures, sections were rinsed 3 × 10 min in PBS followed by 60 min incubation in a blocking solution containing 3% bovine serum albumin (BSA) in PBS with 0.3% Triton X-100 (PBS-Tx). Sections were incubated with the primary antibodies diluted in the blocking solution for 48 h at 4°C, rinsed 3 × 10 min in PBS, and incubated with secondary antibodies diluted in PBS-Tx 24 h at room temperature. Finally, sections were rinsed 3 × 10 min in PBS. To reduce endogenous autofluorescence, monkey sections were immersed in a TrueBlack (Biotium) solution diluted by 70% ethanol for 30 s. The sections were mounted on gelatin-coated slides, and coverslipped using Prolong Diamond Antifade Mountant (Invitrogen #P36961). A series of the adjacent sections were mounted and Nissl-stained with 0.5% Cresyl violet (Acros Organics #AC229630050).

#### Rodents

One week after Fluoro-Gold injection, rodents were deeply anesthetized with an overdose of sodium pentobarbital (100 mg/kg, i.p.) and perfused transcardially with Ringer’s solution (0.85% NaCl, 0.025% KCl, 0.02% NaHCO_3_), followed by 4% paraformaldehyde (PFA) in 0.1M PB. The brains were removed from the skulls, postfixed in the same fresh fixative overnight at 4°C, and put in a cryo-protective solution containing 20% glycerol, 2% DMSO diluted in 0.125M PB at 4°C. The brains were sectioned at 40 μm in the horizontal plane on a freezing microtome and six equally spaced series were collected for further processing.

PCP4+, RE+, and CB+ neurons were visualized using the same antibodies as described above. Sections were rinsed 3 × 10 min in PBS followed by 60 min incubation in a blocking solution containing 5% normal goat serum in PBS-Tx. Sections were incubated with the primary antibodies diluted in the blocking solution for 24 h at 4°C, rinsed 3 × 10 min in PBS-Tx, and incubated with secondary antibodies diluted in PBS-Tx overnight at room temperature. After washes 3 × 10 min in PBS, sections were mounted on gelatin-coated slides, and coverslipped using Entellan new (Merck Millipore #107961).

Coverslipped samples were imaged using an automated scanner (Zeiss, Axio Scan Z1). PCP4, RE, CB, and retrograde labels were thresholded with Zen Blue software (Zeiss), and the labeled-neurons above the threshold were plotted using illustrator software (Adobe). To compare the distribution patterns of labeled neurons, EC was divided into columnar bins by first dividing layer IV into 200 μm-wide bins, and then, extending the columnar bin throughout all layers. The number of immunohistochemically labeled neuron within each layer in each bin was quantified using image J software.^1^ In PCP4-stained sections, this results in 12 parameters for each columnar bin: Number of PCP4+ neurons in layers IIa, IIb, III, Va, Vb, VI, and retrogradely labeled (RTG+) neurons in layers IIa, IIb, III, Va, Vb, VI. The same applies to the RE- and CB-stained sections: number of RE+ neurons in layers IIa, IIb, III, Va, Vb, VI, and CB+ neurons in layers IIa, IIb, III, Va, Vb, VI. Based on these parameters, a principal component analysis was conducted in MATLAB (Mathworks) in order to compare the cytoarchitectonic features of the entorhinal subdivisions. The multiple columnar bins which compose each entorhinal subdivision were used as data points, and the 12 parameters for each columnar bin were used as variables. To perform this analysis, all variables were converted to the proportion of the total number of labeled neurons. To compare the soma size of labeled-neurons, optical sections were obtained with a Zeiss Apotome, and the soma size for each labeled neuron was measured in NeuN images using Zen Blue software. Note that the immunofluorescence for NeuN can be observed in the cytoplasm in addition to the nucleus of a neuron.

### Definition of Subregions and Layer Delineation

Following previous studies on monkey EC (Amaral et al., 1987; Piguet et al., 2018), we cytoarchitectonically divided the monkey EC into seven subdivisions: Eo, the olfactory field of EC; Er, the rostral EC; Elr, the lateral rostral EC; Ei, the intermediate division of the EC; Elc, the lateral caudal EC; Ec, the caudal division of the EC; and Ecl, the caudal limiting division of EC. In addition, we further divided Ei into rostral and caudal subdivisions (Eir and Eic, respectively; Supplementary Figure 1). In contrast to these studies, we changed the definition of the different layers for the monkey EC. Instead of using these original laminar definitions, in this study, we employed a definition that is in line with recently proposed definitions in rodent EC (Sürmeli et al., 2015; Ohara et al., 2018, 2019; Kobro-Flatmoen and Witter, 2019). The prominent differences are the definitions for layer II and layer V. In brief, the original layer II in monkey EC is defined as layer IIa, and the original monkey EC layer V is defined as layer Va in this study. See results for details.

### Statistics

To compare the soma size of labeled-neurons (Supplementary Figure 3), statistical analyses were performed using GraphPad Prism (GraphPad Software) or MATLAB (Mathworks). The details of tests used are described with the results. Group comparisons were made using one-way ANOVA followed by Bonferroni *post hoc* tests to control for multiple comparison. All statistical tests were two-tailed, and thresholds for significance were placed at ^∗^*p* < 0.05, ^∗∗^*p* < 0.01, and ^∗∗∗^*p* < 0.001. All data are shown as mean ± standard errors.

## Results

### Laminar Definition of Monkey Entorhinal Cortex Based on Purkinje Cell Protein 4 Labeling

In previous monkey studies, the entorhinal layers were purely defined by their cytoarchitectonic features (Amaral et al., 1987; Piguet et al., 2018; Witter and Amaral, 2020). In contrast, the existing cytoarchitectonic laminar definition in rodents has recently been complemented by integrating the distribution of molecular and genetic markers that coincide well with traditional cytoarchitecture-based layer definitions (Sürmeli et al., 2015; Ohara et al., 2018, 2019; Kobro-Flatmoen and Witter, 2019). This has facilitated recent detailed functional studies in both MEC (Kitamura et al., 2014, 2015, 2017; Kanter et al., 2017; Rowland et al., 2018; Zutshi et al., 2018) and LEC (Kobro-Flatmoen et al., 2016; Leitner et al., 2016; Vandrey et al., 2020; Lee et al., 2021). Since our aim is to examine the inter-species differences and homology of the EC, it is essential to use the same laminar definition among species. For this purpose, we incorporated the recent definitions of rodents to monkeys by examining the distribution of PCP4. In rodent MEC, PCP4 is a useful marker to identify the layers since PCP4+ neurons mainly distribute in layers III and Vb (Ohara et al., 2018, 2021). The PCP4-negative superficial layer coincides with layer II, and the PCP4-negative deep layer with layer Va. Although PCP4+ neurons are also located in layer VI, this layer can be distinguished from layer Vb by the sparser density of PCP4+ neurons.

**FIGURE 1:**
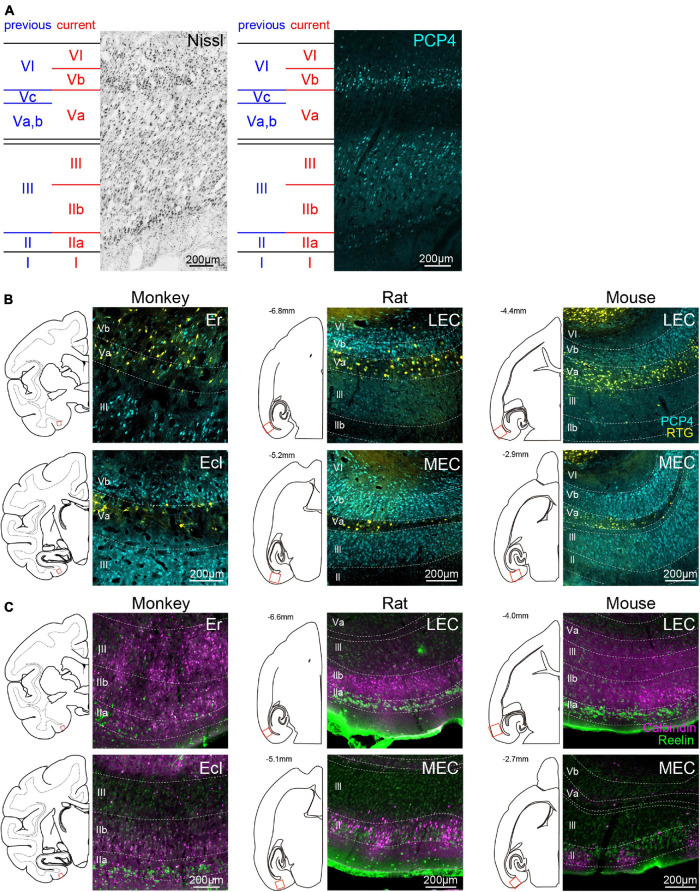
Laminar definition of monkey EC and inter-species comparison of laminar markers. **(A)** Representative Nissl- and PCP4-stained images taken from caudal division of monkey EC (Ec). Previous laminar definition in monkey EC (blue; Amaral et al., 1987), which is based on cytoarchitectonic features, is compared with the definition used in the current study (red). The current definition is based on the distribution of PCP4-labeled neurons (cyan) in addition to the distribution of Nissl-stained cells, and confirmed by connectional data. **(B)** Distribution of PCP4+ neurons (cyan) and NAc-projecting neurons (yellow) in monkeys (left), rats (middle), and mice (right). Fluorescent images were taken from monkey Er (top-left) and Ecl (bottom-left) in coronal sections, and rodent LEC (top-middle, top-right) and MEC (bottom-middle, bottom-right) in horizontal sections corresponding to the boxed areas in the line drawings. For visual purpose, the images of monkey EC are horizontally flipped. **(C)** Distribution of CB+ neurons (magenta) and RE+ neurons (green) in the EC of monkeys and rodents. The organization of the panels are the same as **(B)**.

Similar to the case in rodents, the distribution patterns of PCP4+ neurons in monkey EC showed a marked laminar pattern (Figure 1A). *Layer I* was a cell poor layer beneath the pia which was immunonegative to PCP4. In previous monkey studies, “layer II” was defined as a thin layer of darkly Nissl-stained cells, and “layer III” as the deeper layer separated from “layer II” by a narrow cell-free zone (Amaral et al., 1987; Piguet et al., 2018; see Figure 1A, blue labels). Following that nomenclature, “layer II” showed low PCP4 staining, and the intensity of PCP4 staining in “layer III” indicated a further division into a superficial layer with low PCP4 labeling and a deep layer with high PCP4 labeling. Based on the fact that in rodents the PCP4 expression level is higher in layer III cells than in layer II cells (Ohara et al., 2018, 2021), we defined the deeper PCP4+ “layer III” as *layer III* (Figure 1A, red labels). The original “layer II” and superficial “layer III” (Figure 1A, blue labels), which show relatively low PCP4 labeling, will in this study be referred to as *layer IIa* and *layer IIb*, respectively (Figure 1A, red labels). The definition of *layer IIa* was further supported by the specific expression of RE (details in subsequent section “Distributions of RE- and CB-Positive Neurons,” Figure 1C). Deep to the cell-sparse zone in between layers III and V, the *lamina dissecans*, we observed a PCP4-negative layer composed of pyramidal cells. In a Nissl stain it was apparent that within this PCP4-negative layer both the cell size and cell density varied along the radial axis. Superficially, the layer contained mainly large pyramidal neurons and in the deeper zone, neurons were smaller and showed a lower density. This PCP4-negative layer is followed by a strikingly densely packed layer of small pyramidal neurons, many of which show high expression levels of PCP4. Continuing along the radial axis toward the white matter, there is a zone that expressed lower levels of PCP4 expression. In the original nomenclature, the PCP negative zone was referred to as “layer V,” which was further subdivided into a superfical Va and Vb, mainly comprising large pyramidal neurons that are quite dispersed, and a more cell sparse zone with smaller pyramidal neurons, referred to as layer Vc (Amaral et al., 1987; Figure 1A, blue labels). According to the nomenclature of the latter authors, the deeper PCP4+ lamina should be referred to as “layer VI.” Following the recent definition in rodents (Sürmeli et al., 2015; Ohara et al., 2018), layer Va is composed of sparsely distributed large pyramidal cells which are immunonegative for PCP4, whereas layer Vb is made up of densely packed smaller cells that are immunopositive for PCP4. Following this definition in rodents, we here defined the PCP4-negative layer, including “layers Va, Vb, and Vc” according to the previous definition, as layer Va (Figure 1A, blue vs. red labels). The PCP4+ layer, which was composed of densely packed cells smaller than layer Va, we defined as layer Vb; this layer was originally considered as part of “layer VI.” In the current definition, layer VI comprises small PCP4+ cells but the density of these cells is much sparser than in layer Vb. Although the distribution of PCP4+ neurons differed along the rostrocaudal axis (see details in subsequent results), the layers were defined accordingly across all subdivisions by using both the molecular and cytoarchitectonic features (Supplementary Figure 1). In the following sections, we will use this updated laminar definition of monkey EC to identify the inter-species differences and similarities between monkeys and rodents (Figures 1B,C).

### Distributions of Purkinje Cell Protein 4-Positive Neurons and Subcortical Projecting Neurons

**FIGURE 2:**
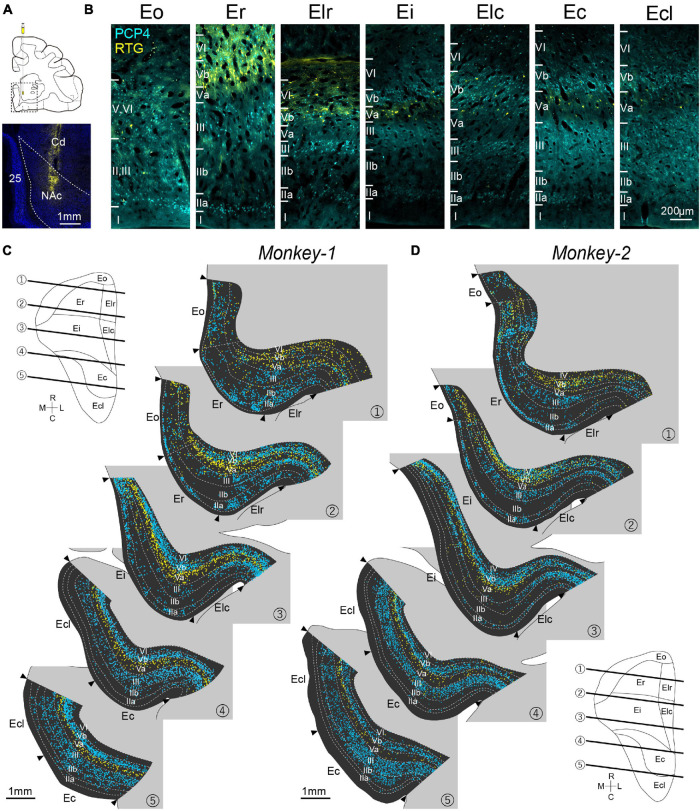
Distribution of PCP4+ neurons and NAc-projecting neurons in monkey EC. **(A)** Injection site of the retrograde AAV in the NAc. 25: area 25; Cd: Caudate. **(B)** Fluorescent micrographs of retrogradely labeled (RTG+) NAc-projecting neurons (yellow) and PCP4+ neurons (cyan) in EC subdivisions. **(C,D)** Series of coronal sections of EC showing the distribution of NAc-projecting neurons (yellow plot) and PCP4+ neurons (cyan plot) in Monkey-1 **(C)** and Monkey-2 **(D)**. NeuN-labeling was used to delineate the individual layers and the EC subdivision. The approximate rostrocaudal levels of the sections are indicated in the unfolded map of EC.

**FIGURE 3:**
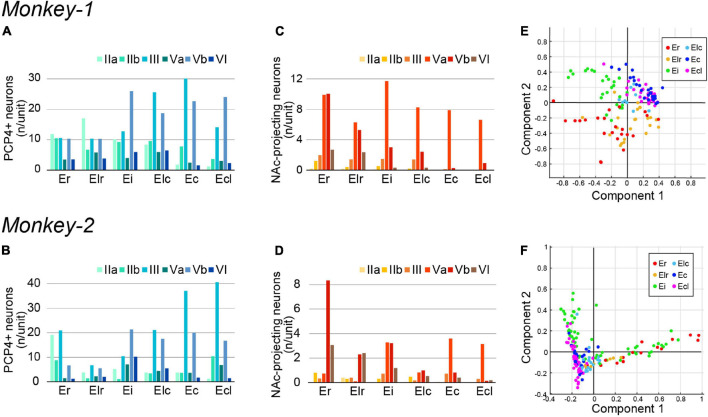
Quantification of PCP4+ neurons and NAc-projecting neurons in monkey EC. **(A–D)** A diagram showing the number/unit of PCP4+ neurons **(A,B)** and NAc-projecting neurons **(C,D)** in each EC subdivision across layers in Monkey-1 **(A,C)** and Monkey-2 **(B,D)**. **(E,F)** Principal component analysis based on the parameters show in **(A–D)** (see section “Materials and Methods” for detail). **(E)** The two principal components explaining most of the variance in case of Monkey-1, with the combined information of NAc-projecting neurons in layer Va/Vb contributing most. **(F)** The two principal components explaining most of the variance in case of Monkey-2, with component 1 mainly representing the number of NAc-projecting neurons in layer Vb whereas component 2 mainly represents the number of NAc-projecting neurons in layer Va.

Recent studies in rodents reported that molecularly defined sublayers of entorhinal layer V show distinct connectivity (Sürmeli et al., 2015). To examine whether the monkey EC share such connectionally defined laminar organization, orange fluorescent protein-expressing retrograde AAV (AAV2Retro-hSynI-mKO2) was injected into NAc (Figure 2A), which has been known to receive massive inputs from layer Va neurons of both MEC and LEC in rodents (Sürmeli et al., 2015; Ohara et al., 2018). The distribution of retrogradely labeled neurons was examined together with the immunoactivity for PCP4 in EC ipsilateral to the injection site (Figure 2B). To systematically examine the labeling patterns across layers and subdivisions, we divided the EC into columnar bins, and quantified the number of labeled-neurons (see section “Materials and Methods”). Due to the poor laminar organization, Eo was excluded from this quantification. In both subjects, retrogradely labeled (RTG+) neurons were observed throughout the whole EC. In caudal EC, comprising Ec and Ecl, the labeling patterns for both PCP4 and RTG were similar to that reported in the rodent MEC. Immunoreactivity for PCP4 was present in layers III and Vb, whereas NAc-projecting neurons were mainly present in layer Va (Figures 2B–D, 3A–D). Similar to rodents (Ohara et al., 2018, 2021), the number of PCP4+ neurons in layer III was lower in rostral than in caudal subdivisions, and this decrease was accompanied by an increase of PCP4+ neurons in layer IIa (Figures 3A,B). In addition, the number of PCP4+ neurons in layer Vb decreased in rostral sections. Surprisingly, the distribution of NAc-projecting neurons within the deep layers also differed along the rostrocaudal axis, such that the NAc-projecting neurons, which were predominantly located in layer Va in caudal sections, are shifted to a preferred deeper location in rostral sections of both monkeys (Figures 3C,D). Such differences were striking in Er and Elr of Monkey-2, showing that retrogradely labeled-neurons were distributed prominently in layer Vb and VI (Figures 2D, 3D). Note that in Monkey-1, this trend for NAc projecting neurons to occupy layer Vb is present but less apparent, still having some retrogradely labeled neurons in layer Va of Er and Elr (Figures 2C, 3C). To confirm whether projection patterns to other telencephalic structures are similar, we injected retrograde AAV also into another telencephalic subcortical structure, AMG (Supplementary Figure 2). We recently reported in rodents that AMG not only receive inputs from layer Va neurons in LEC and MEC, but also from LEC layer IIb neurons (Ohara et al., 2019). Similar to that, in the monkey, AMG-projecting neurons were observed in the superficial layers at the rostral level including Eo and Er, which was different from the distribution of the NAc-projecting neurons. In the deep layers, the labeling patterns were consistent with the results of NAc injected samples such that neurons projecting to telencephalic subcortical structures intermingle with PCP4+ neurons in layer Vb in rostral parts of monkey EC. This largely differs from layer V organization in rodents, which shows a clear segregation of PCP4+ layer Vb and the subcortical-projecting neurons in layer Va throughout the extent of EC (Figure 1B). Similar to the pattern seen in rodents (Sürmeli et al., 2015), NAc-projecting neurons were typical pyramidal cells with significantly larger soma size compared to the PCP4+ layer V neurons in both rostral and caudal EC, and there were no significant differences in the soma size of each cell type between the rostral and caudal EC (Supplementary Figure 3A, Er PCP4+ neurons, 185.9 ± 6.8; Er RTG+ neurons, 278.3 ± 10.4; Ec PCP4+ neurons, 170.6 ± 2.7; Ec RTG+ neurons, 262.7 ± 9.2 μm^2^; *p* < 0.001 for Er PCP4+ neurons vs. Ec PCP4+ neurons and Er RTG+ neurons vs. Ec RTG+ neurons, one-way ANOVA followed by Bonferroni’s multiple comparison test). In addition, despite the mixed distribution of PCP4+ neurons and retrogradely labeled neurons in the rostral EC, retrogradely labeled neurons that showed PCP4+ colabeling were hardly observed (Er 0%; Elr 0%; Ei 0.39%; Elc 0.66%; Ec 1.39%; Ecl 0.51%). These results indicate that monkey EC layer V is composed of two cell types with distinct molecular identity, soma size, and connectivity, which intermingle in the rostral subdivisions and segregate in different sublayers in the caudal subdivisions.

To examine whether the EC subdivisions can be distinguished based on the above cell-type specific markers, we conducted principal component analyses based on the number of PCP4+ neurons and the NAc-projecting neurons across layers. This resulted in a moderate separation between the caudal EC, that is Ec and Ecl, and the rostral EC, that is Er and Elr, in both subjects (Figures 3E,F). Interestingly, Ei showed properties of both caudal and rostral EC subdivisions in Monkey-2.

### Distributions of RE- and CB-Positive Neurons

**FIGURE 4:**
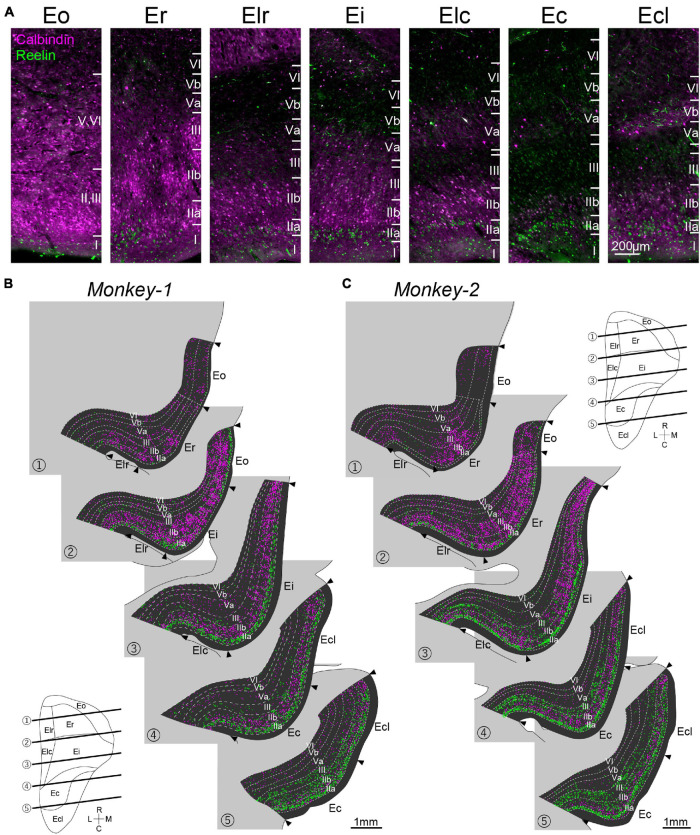
Distribution of CB+ and RE+ neurons in monkey EC. **(A)** Fluorescent images of CB+ neurons (magenta) and RE+ neurons (green) in EC subdivisions. **(B,C)** Series of coronal sections of EC showing the distribution of CB+ neurons (magenta) and RE+ neurons (green) in Monkey-1 **(B)** and Monkey-2 **(C)**. NeuN-labeling was used to delineate the individual layers and the EC subdivision. The approximate rostrocaudal levels of the sections are indicated in the unfolded map of EC.

**FIGURE 5:**
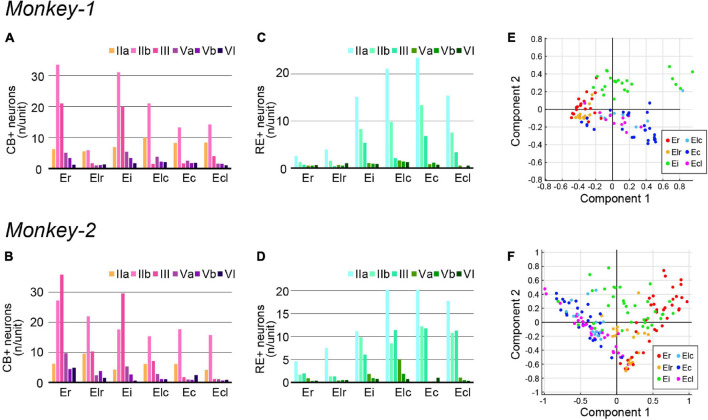
Quantification of CB+ and RE+ neurons in monkey EC. **(A–F)** A diagram showing the number/unit of CB+ neurons **(A,B)** and RE+ neurons **(C,D)** in each EC subdivision across layers in Monkey-1 **(A,C)** and Monkey-2 **(B,D)**. **(E,F)** Principal component analysis based on the parameters shown in **(A–D)** (see section “Materials and methods” for detail). The plot shows the first two principal components, with both component 1 and 2 mainly representing the combined information from the five variables, CB+ neurons in layer IIb and III, RE+ neurons in layer IIa, IIb, and III, in Monkey-1 **(E)** and Monkey-2 **(F)**.

The distributions of the molecular markers for EC layer II, RE, and CB, were examined in monkey EC, contralateral to the AAV-injection site (Figure 4A). Similar to the organization of LEC layer II in rodents, RE+ neurons were present in layer IIa, whereas the CB+ neurons mainly distributed in layer IIb (Figure 4A). This segregation between layer IIa and IIb continued throughout the whole extent of EC, and the two cell-types did not intermingle in layer II even in the caudal sections which clearly differs from that in rodent MEC where the two cell-types intermingle in layer II (Figure 1C). Particularly in the rostral subdivisions, RE+ layer IIa cells were separated by relatively cell sparse zones, and formed cell islands, as previously reported (Amaral et al., 1987). In addition to layer II, RE+ and CB+ neurons distributed also in layer III, which was especially striking in Monkey-2. The number of labeled neurons in layer II and III largely differed along the rostrocaudal axis (Figures 4B,C), and the CB+ neurons increased in the rostral subdivisions (Figures 5A,B) which is in line with previous study (Suzuki and Porteros, 2002). In contrast, the number of RE+ neurons increased in the caudal subdivisions in both layer II and III (Figures 5C,D). The soma size of the RE+ layer III neurons was similar to that of the RE negative (RE-) layer III neurons, and was significantly smaller than the RE+ layer IIa neurons in Ec and Ecl (Supplementary Figure 3B, layer IIa RE+ cells, 374.7 ± 6.0; layer III RE+ cells, 208.4 ± 4.0; layer III RE- cells, 193.8 ± 5.1; *p* < 0.001 for layer IIa RE+ cells vs. layer III RE+ cells and layer IIa RE+ cells vs. layer III RE- cells, one-way ANOVA followed by Bonferroni’s multiple comparison test).

One prominent feature of CB+ neurons in layer II is that they form periodic clusters, which have been confirmed both in rodent MEC and in human caudal EC (Kitamura et al., 2014; Ray et al., 2014; Naumann et al., 2016). In contrast to these reports, such calbindin patches were observed not in the caudal EC but in Er and Ei (Figures 4A–C). In addition, the calbindin patches were not confined to layer IIb but extended into layer III, which is in line with a previous report (Suzuki and Porteros, 2002). We also noticed that the distribution of CB+ neurons in Er was not homogeneous, such that they are more numerous in the medial area, close to the border with Eo, than in the lateral area (Figures 4B,C).

Principal component analysis based on the number of RE+ and CB+ neurons across layers resulted in a moderate separation between the caudal EC, that is Elc, Ec, and Ecl, and the rostral EC, that is Er and Elr (Figures 5E,F). Ei, however, showed a feature different from other EC subdivisions in both subjects, and in case of Monkey-2, Ei shared properties with the rostral and caudal EC. This was less apparent in Monkey-1.

### Parcellation of Ei Based on Molecular Markers

Principal component analysis shown in Figures 3E,F, 5E,F indicate that Ei may comprise diverse subregions. Indeed, previous anatomical studies indicated that Ei may be subdivided into a rostral and a caudal portion based on the projection patterns of inputs from the presubiculum (Witter and Amaral, 2020). To examine whether the labeling patterns differ within Ei, the distribution of labeled-neurons in Ei were quantified in the rostral portion of Ei (Eir) and the caudal portion of Ei (Eic).

**FIGURE 6:**
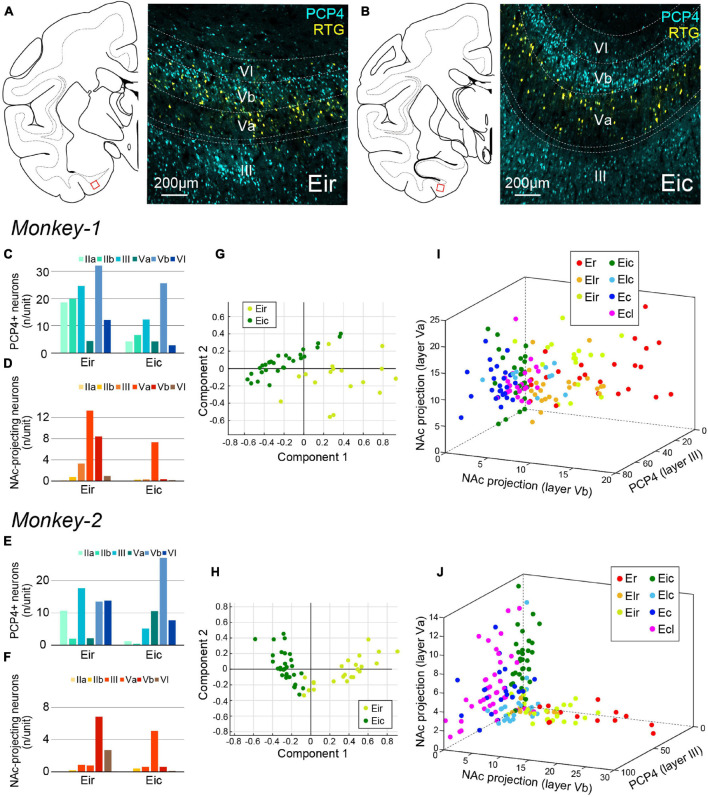
Parcellation of Ei based on PCP4+ and NAc-projecting neurons. **(A,B)** Fluorescent micrographs of NAc-projecting retrogradely labeled neurons (RTG+; yellow) and PCP4+ neurons (cyan) in Eir **(A)** and Eic **(B)**. For both images, the position is indicated as the red squared area in the schematic line drawing of the coronal section to the left. **(C–F)** A diagram showing the number/unit of either PCP4+ neurons **(C,E)** or NAc-projecting neurons **(D,F)** in Eir and Eic across layers in Monkey-1 **(C,D)** and Monkey-2 **(E,F)**. **(G,H)** Principal component analysis for Monkey-1 **(G)** and Monkey-2 **(H)** based on the parameters shown in **(C–F)**. **(I,J)** Separation of EC subdivisions using number/unit of NAc-projecting neurons in layer Va, and layer Vb, and the number of PCP4+ neurons in layer III as distinction criteria, in Monkey-1 **(I)** and Monkey-2 **(J)**.

In sections immunostained for PCP4, clear distribution differences of NAc-projecting neurons were observed in layer V between Eir and Eic. In Eic, NAc-projecting neurons were localized in layer Va whereas in Eir, they were also present in layer Vb (Figures 6A–F). In addition to these differences in layer V, there were more layer III PCP4+ neurons in Eir than in Eic. Principal component analysis based on the number of PCP4+ and NAc-projecting neurons resulted in a separation between Eir and Eic (Figures 6G,H). The most prominent features aiding to separate the EC subdivisions were thus “the number of retrogradely labeled neurons in layer Va,” “the number of retrogradely labeled neurons in layer Vb,” and “the number of PCP4+ neurons in layer III,” which indicates that Eir share properties with the rostral subdivisions whereas Eic is closer to the caudal subdivisions (Figures 6I,J).

**FIGURE 7:**
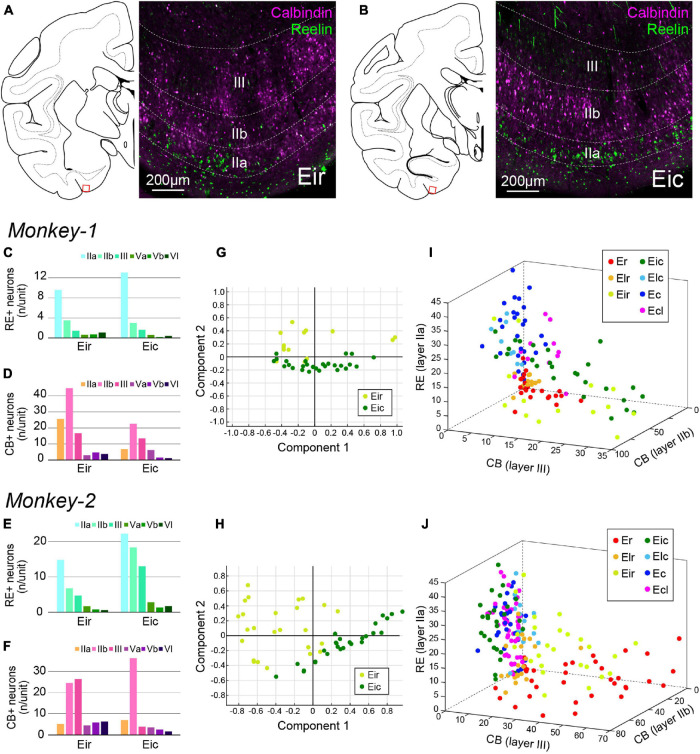
Parcellation of Ei based on CB+ and RE+ neurons. **(A,B)** Fluorescent micrographs of CB+ neurons (magenta) and RE+ neurons (green) in Eir **(A)** and Eic **(B)**. For both images, the position is indicated as the squared area in the schematic line drawing of the coronal section to the left. **(C–F)** A diagram showing the number/unit of either RE+ neurons **(C,E)** or CB+ neurons **(D,F)** in Eir and Eic across layers. **(G,H)** Principal component analysis for Monkey-1 **(G)** and Monkey-2 **(H)** based on the parameters shown in **(C–F)**. **(I,J)** Separation of EC subdivisions using number/unit of CB+ neurons in layer IIa, and layer III, and the number of RE+ neurons in layer IIa as distinction criteria, in Monkey-1 **(I)** and Monkey-2 **(J)**.

In sections immunostained for CB and RE, differences were mainly observed in the superficial layers especially for the CB-labeling. CB+ neurons tend to cluster in patches in Eir compared to Eic, while the islands of RE+ neurons were more prominent in Eir than in Eic (Figures 7A,B). Although the distribution patterns of RE+ and CB+ neurons were inconsistent between the two subjects (Figures 7C–F), principal component analysis showed a separation between Eir and Eic in both subjects (Figures 7G,H). In Monkey-2, Eir and Eic shared similar properties with rostral- and caudal-subdivisions, respectively, based on the “the number of CB+ neurons in layer IIb,” “the number of CB+ neurons in layer III,” and “the number of RE+ neurons in layer IIa” (Figure 7J). This, however, was not clear in Monkey-1 (Figure 7I).

## Discussion

### Homolog of Medial and Lateral Entorhinal Cortex in Primates

Our understanding of the EC has progressed substantially by studies in rodents focusing on the functionally distinct subregions, MEC that processes spatial information and LEC that represents the time and content of episodes (Hafting et al., 2005; Deshmukh and Knierim, 2011; Xu and Wilson, 2012; Tsao et al., 2018, 2013; Montchal et al., 2019). To apply the improved knowledge of rodent EC to primates, it would be advantageous to define the homolog of rodent MEC and LEC in primates. One useful landmark to differentiate MEC from LEC is the input to MEC from the presubiculum, as reported in several non-primate mammalian species (Witter et al., 2017). In monkeys, presubicular inputs preferentially target the caudal portion of EC, which includes Elc, Ec, and Ecl. In addition, presubicular fibers are located in the caudal portion of Ei (Witter and Amaral, 2020). This indicates that the monkey homolog of the non-primate MEC does not coincide with the current EC parcellation, which is based on cytoarchitectonic features, and that Ei may has to be further divided along the rostrocaudal axis.

In this study, we focused on cell-type-specific markers and found that Ei can indeed be further divided into rostral and caudal portions on the basis of the distribution of PCP4+ neurons and telencephalon-projecting neurons in layer V. The caudal Ei (Eic) showed similarity to the caudal EC subdivisions, that is, Elc, Ec, and Ecl, showing a clear segregation in the distribution of the two cell types in layers Va and Vb. In contrast, the rostral Ei (Eir) was found to be more similar to the rostral EC subdivisions, that is, Er and Elr, with the two cell types being intermingled in both layers Va and Vb. Although it is unclear whether this cell-type-based border corresponds to the terminal distribution pattern of projections from the presubiculum, we can extrapolate, based on the specific cell marker expression patterns shown here, that the caudal subdivisions including Eic are homologous to rodent MEC, while the rostral subdivisions including Eir are homologous to rodent LEC. In the following sections, we tentatively call the two subdivisions monkey MEC and monkey LEC, respectively.

### Laminar Organization of Entorhinal Cortex in Primates

The laminar definition of EC has recently been reconsidered based on the distribution of molecularly defined neurons in rodents (Varga et al., 2010; Ramsden et al., 2015; Sürmeli et al., 2015; Kobro-Flatmoen and Witter, 2019; Ohara et al., 2019). To promote translational research of EC, we implemented this recent definition to study the laminar organization of EC in primates instead of using the purely cytoarchitectonic laminar definition (Amaral et al., 1987; Piguet et al., 2018), and integrated these chemoarchitectonic criteria with connectional data. In brief, the rodent data show the following laminar definition (for review, see Nilssen et al., 2019). *Layer IIa* is a thin layer of RE+ stellate cells which project to the dentate gyrus and CA3, whereas *layer IIb* comprises CB+ pyramidal cells that only show minor hippocampal projections and widespread though sparse telencephalic cortical and subcortical projections. *Layer III* neurons express PCP4 and project to CA1 and the subiculum. *Layer V* is composed of *layer Va* and *layer Vb*, which mediate the hippocampal information to telencephalic structures. Large PCP4-negative pyramidal cells located in *layer Va* are the major origin of telencephalic cortical and subcortical projections, whereas small PCP4+ neurons are densely packed in *layer Vb* and are among the recipients of hippocampal outputs from CA1 and the subiculum.

The present data indicate that the laminar organization of monkey EC displays a strong similarity to that of rodent EC with some exceptions. In monkey MEC, the cell-type-based laminar organization was found to be similar to that in rodent MEC: PCP4+ neurons were distributed in layers III and Vb, whereas the neurons projecting to telencephalic structures, as tested with projections to NAc and AMG, were localized in layer Va. Since the soma size of PCP4+ layer Vb neurons is small whereas that of telencephalon-projecting neurons in layer Va is large, the border between layers Va and Vb is also apparent in Nissl-stained sections. The only clear difference between monkey and rodent MEC was the labeling pattern in layer II. The organization of layer II of monkey MEC was rather similar to that of rodent LEC, in that there was a clear segregation between the RE+ layer IIa and the CB+ layer IIb. In addition, CB+ patches were hardly observed in monkey MEC, unlike rodent MEC (Suzuki and Porteros, 2002; Kitamura et al., 2014; Ray et al., 2014; Naumann et al., 2016).

In monkey LEC, the cell-type-based laminar differentiation between superficial layers was similar to that in rodent LEC: RE+ and CB+ neurons were found to be confined to layers IIa and IIb, respectively, and the PCP4 expression level of layer III neurons was lower than that in monkey MEC. The RE+ layer IIa neurons were separated by cell-sparse zones to form cellular islands. One exception is the presence of CB+ patches in Er and rostral Ei, which is in line with a previous report (Suzuki and Porteros, 2002). The most striking difference between monkeys and rodents was the organization of layer V in monkey LEC. In rodent LEC, neurons projecting to telencephalic structures were specifically localized in layer Va but not in layer Vb, which is also the case in rodent and monkey MEC. In monkey LEC, however, such telencephalon-projecting neurons were distributed in both layers Va and Vb. Although the projection neurons were intermingled in layer Vb, there was no overlap between RTG+ and PCP4+ neurons. We thus conclude that the two populations are almost completely segregated. Owing to the mixed distribution of the two cell types, the border between layers Va and Vb in the Nissl-stained sections was more obscure in monkey LEC than in monkey MEC.

### Layer V Entorhinal Cortex Circuits in Rodents and Primates

In recent studies, the circuits of entorhinal layer V in rodents were examined, which revealed how the hippocampal information is sent out to telencephalic structures. The main recipients of dorsal hippocampal projections in EC are PCP4+ layer Vb neurons (Sürmeli et al., 2015; Rozov et al., 2020). These layer Vb neurons are locally projecting excitatory neurons that mediate the following two circuits in the hippocampal memory system: (1) a circuit of hippocampal outputs to telencephalic areas through projections to layer Va and (2) a feedback projection sending information back to the EC-hippocampal loop *via* neurons in layers II and III (Ohara et al., 2018). Interestingly, the intrinsic connections of layer Vb neurons differ between LEC and MEC in that the layer Vb-to-Va hippocampal output circuit seems more prominent in LEC than in MEC (Ohara et al., 2021). These differences in layer Vb circuits between LEC and MEC indicate that LEC might be a more relevant player in mediating the hippocampal-cortical interplay that is necessary for systems memory consolidation (Buzsáki, 1996; Frankland and Bontempi, 2005; Eichenbaum et al., 2012).

In this study, we have shown that monkey entorhinal layer V is also composed of PCP4+ neurons and neurons projecting to telencephalic structures. Although detailed circuit analysis of specific cell types has not yet been carried out in monkey EC, in a previous anterograde tracing study, the intrinsic connections of EC were examined using chemical anterograde tracers (Chrobak and Amaral, 2007). These authors have reported that in monkey MEC, the intrinsic projections from layer VI (redefined as layer Vb in this study) to superficial layers avoid layer V (redefined as layer Va in this study, see Figure 2B’ in Chrobak and Amaral, 2007), and thus are in line with our data in rodents (Ohara et al., 2021). On the basis of these similarities, we assume that the intrinsic circuits of MEC layer V are conserved between rodents and monkeys, and favors the EC-hippocampal loop rather than the hippocampal-telencephalic output circuit.

In contrast to MEC, the laminar organization of monkey LEC layer V largely differed from that of rodent LEC, and PCP4+ neurons and telencephalon-projecting neurons did not obey the rodent laminar arrangement but were intermingled in layer V. This raises the possibility that the hippocampal projection may directly innervate the telencephalon-projecting neurons in monkey LEC, and therefore, the monkey LEC mediates the transfer of hippocampal information to telencephalic structures more efficiently than the rodent LEC. It, however, must be noted that despite the mixed distribution, PCP4 and retrograde labeling were hardly colocalized in single neurons in monkey LEC layer V. This points out the other possibility that regardless of the different laminar distribution, LEC layer V neurons are connectionally similar and are embedded within similar circuits, both in rodents and monkeys. To further understand the organization of entorhinal layer V, it is essential to identify the targets of the hippocampal outputs in monkeys. The hippocampal-entorhinal projection has only been examined in detailed in rodents, reporting that the dorsal hippocampal neurons innervate entorhinal layer Vb but not Va (Sürmeli et al., 2015; Ohara et al., 2018). Interestingly, a recent study in rodents has shown that hippocampal projections originating from a more ventral level synapse on layer Va in addition to layer Vb neurons (Rozov et al., 2020), indicating that the hippocampal-entorhinal circuit may be more complicated than initially reported. In monkeys, although the termination of the hippocampal projections in entorhinal deep layers has been reported, layer V sublayers were not taken into account (Rosene and Van Hoesen, 1977; Witter and Amaral, 2020). Identifying the specific targets of these projections will enable us to understand whether the hippocampal-cortical output circuit might be more prominent in monkey LEC than in monkey MEC, as suggested in rodents (Ohara et al., 2021).

A developmental study in mice reported data suggesting that MEC is derived from the medial pallium similarly to the hippocampus, whereas LEC is derived from a distinct dorsolateral caudal pallial sector (Medina et al., 2017). This difference in the embryonic origin may on the one hand explain the rather conserved organization of the hippocampus and MEC during the course of the mammalian evolution. On the other hand, LEC may have increased its complexity in primates together with the neocortex. To allow for evidence-based translation from animal experimental data to human application, the cell-type-based organization of monkey LEC reported here points to the importance of studying the entorhinal organization not only in rodents, but also in monkeys. Recent advances in viral vectors that enable targeting specific neurons are likely essential for subsequent studies. These include viral tools targeting neurons with specific projections (Inoue et al., 2015; Oyama et al., 2021), or specific interneurons in non-human primates (Dimidschstein et al., 2016; Vormstein-Schneider et al., 2020; Mich et al., 2021), as well as enhancer-based viral vectors targeting entorhinal excitatory cells (Nair et al., 2020). The cell-type-based global map provided in this study will accelerate the application of these genetic tools in monkeys for better understanding of the role of EC in memory and navigation and will further the translational potential of the experimental observations in monkeys.

## Conclusion

The laminar organization of layer V, as defined by the cell types, is different between the rostral and caudal EC in monkeys. These rostral and caudal portions of EC likely correspond to LEC and MEC in rodents, respectively. In addition, we found that a clear laminar arrangement of layer V neurons, which is evident in rodent LEC, is absent in the rostral EC of monkeys. These observations suggest that LEC in primates may have increased its complexity through evolution, and developed into a uniquely tuned information processing system, serving the efficient transfer of hippocampal output to cortical and subcortical structures. Further investigations are required to fully appreciate the potential relevance of the primate LEC.

## Data Availability Statement

The raw data supporting the conclusions of this article will be made available by the authors, without undue reservation.

## Ethics Statement

The animal study was reviewed and approved by the Animal Welfare and Animal Care Committee of the Primate Research Institute, Kyoto University and the Center for Laboratory Animal Research of Tohoku University.

## Author Contributions

SO and MPW conceived the study design. K-II produced the AAV vectors. KK, ST, AZ, and RY collected the experimental samples in monkeys, both under supervision of MT. TK collected the samples in rodents. RY, TK, and SO analyzed the data. All authors contributed to the discussions that resulted in the current manuscript, which was written by SO and RY, and edited by MPW, K-IT, and MT. All authors approved the final version of the manuscript.

## Conflict of Interest

The authors declare that the research was conducted in the absence of any commercial or financial relationships that could be construed as a potential conflict of interest.

## Publisher’s Note

All claims expressed in this article are solely those of the authors and do not necessarily represent those of their affiliated organizations, or those of the publisher, the editors and the reviewers. Any product that may be evaluated in this article, or claim that may be made by its manufacturer, is not guaranteed or endorsed by the publisher.

## Footnotes

1
http://rsb.info.nih.gov/ij
