# Supplementary material for: Laminar Organization of the Entorhinal Cortex in Macaque Monkeys Based on Cell-Type-Specific Markers and Connectivity
Source: Front Neural Circuits. 2021 Dec 7;15:790116. doi: 10.3389/fncir.2021.790116 (PMC8688913; doi:10.3389/fncir.2021.790116)
Supplement: Supplementary file 1 [file Data_Sheet_1.PDF]

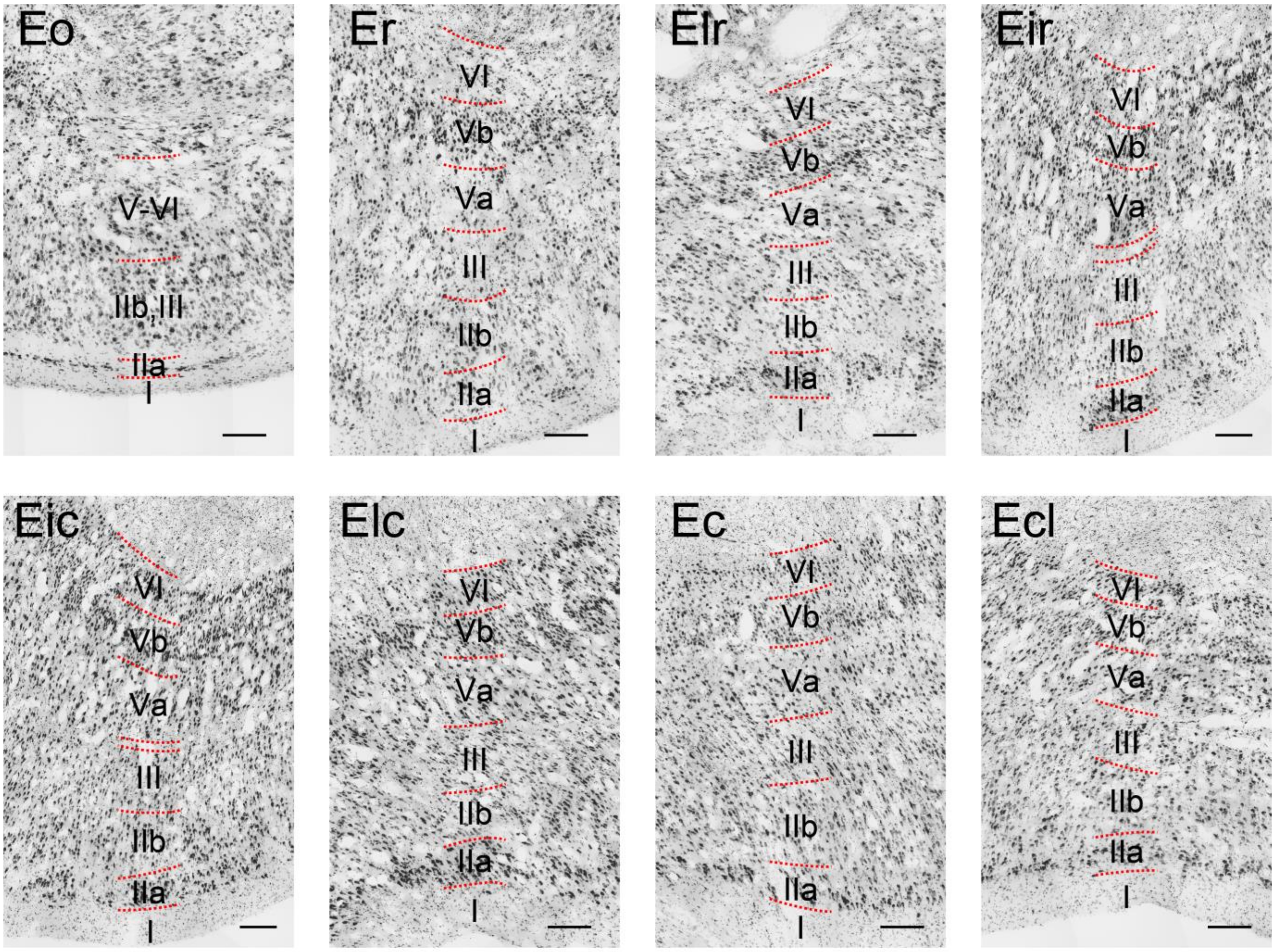

Supplementary Figure 1. Photomicrographs of the Nissl stained coronal sections of the monkey EC. Nissl images are arranged from the rostral to the caudal subdivisions. Roman numbers separated by red dotted lines indicate individual cortical layers and their boundaries. The features of each layer are described in the Results (section 3.1). Eo, olfactory field; Er, rostral field; Elr, lateral rostral field; Eir, intermediate rostral field; Eic, intermediate caudal field; Elc, lateral caudal field; Ec, caudal field; Ecl, caudal limiting field. Scale bars represent 200 µm.

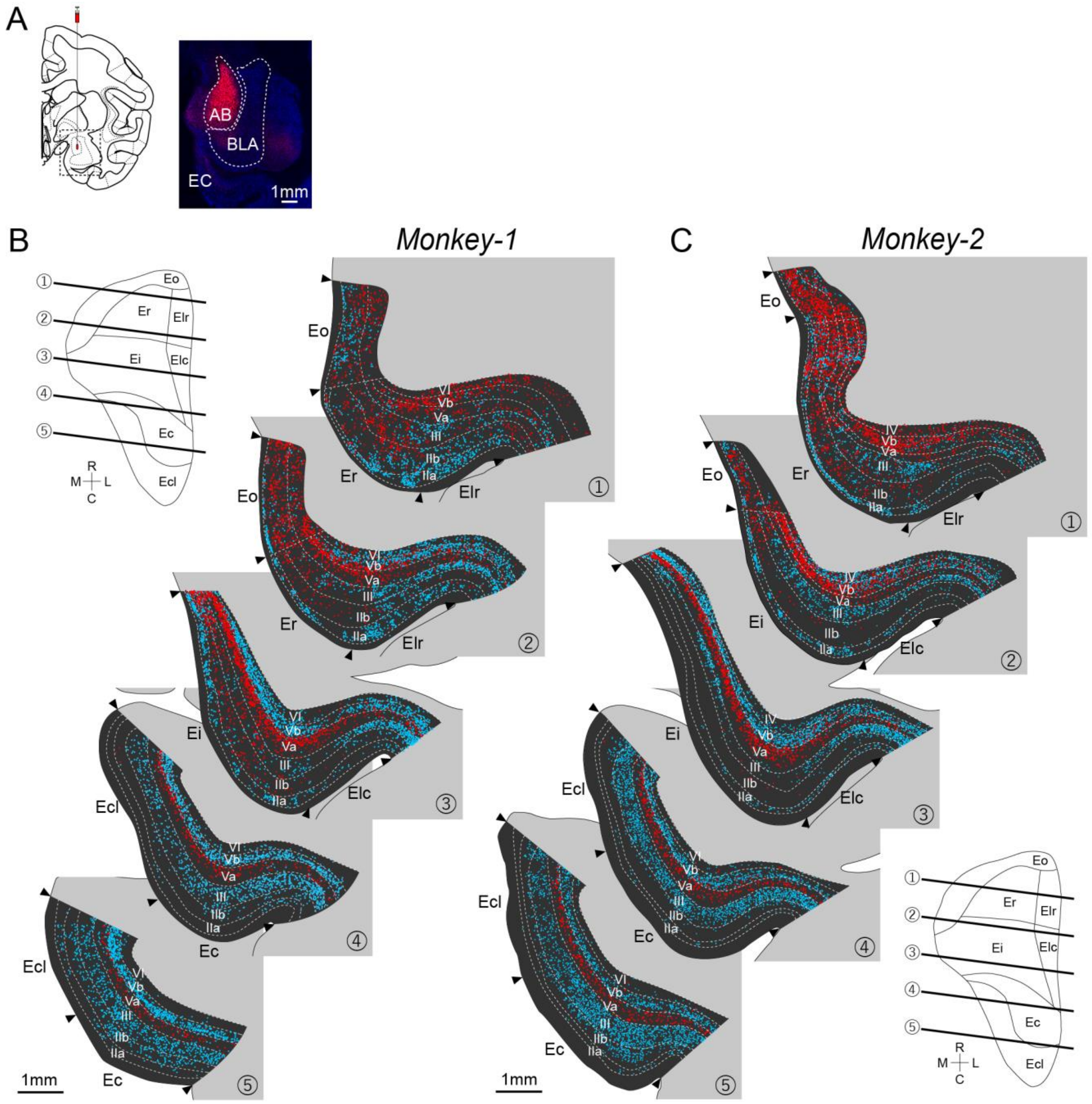

Supplementary Figure 2. Distribution of PCP4+ neurons and Amygdala (AMG)-projecting neurons in monkey EC. (A) Injection site of the retrograde AAV in the AMG. AB: accessory-basal nucleus of amygdala; BLA: basolateral amygdala. (B, C) Series of coronal sections of EC showing the distribution of RTG+ neurons (red) and PCP4+ neurons (cyan) in Monkey-1 (B) and Monkey-2 (C). NeuN-labeling was used to delineate the individual layers and the EC subdivision. The approximate rostrocaudal levels of the sections are indicated in the unfolded map of EC. The distribution of the AMG-projecting neurons was similar to that of the NAc-projecting neurons (Figure 2) in the deep layers. In addition to the deep layers, the AMG-projecting neurons also distributed in the superficial layers at the rostral level including Eo and Er, which was different from the distribution of the NAc-projecting neurons.

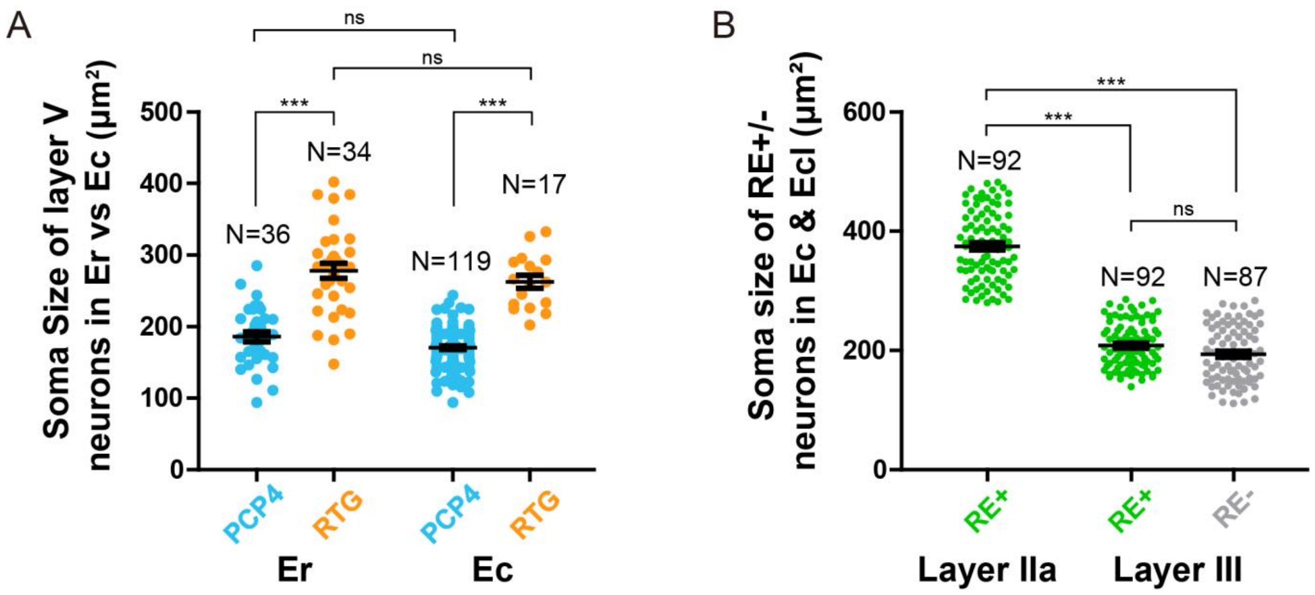

Supplementary Figure 3. Soma size of the molecularly defined entorhinal neurons. (A) Soma size of the PCP4+ and RTG+ NAc projecting layer V neurons in Er and Ec of Monkey-1 (error bars: mean ± standard errors; one-way ANOVA, $F_{3,202}$ = 85.95, p<0.0001, Bonferroni's multiple comparison test, ***p<0.001). (B) Soma size of the RE+ layer IIa neurons and RE+ and RE- layer III neurons in EC and Ecl of Monkey-1 (error bars: mean ± standard errors; one-way ANOVA, $F_{2,268}$ = 392.1, p=0.0005, Bonferroni's multiple comparison test, ***p<0.001).
